# Healthcare utilization and productivity loss in glioma patients and family caregivers: the impact of treatable psychological symptoms

**DOI:** 10.1007/s11060-020-03454-3

**Published:** 2020-03-14

**Authors:** Florien W. Boele, David Meads, Femke Jansen, Irma M. Verdonck-de Leeuw, Jan J. Heimans, Jaap C. Reijneveld, Susan C. Short, Martin Klein

**Affiliations:** 1 Leeds Institute of Medical Research at St James’s, St James’s University Hospital, Leeds, LS9 7TF UK; 2 Faculty of Medicine and Health, Leeds Institute of Health Sciences, University of Leeds, Leeds, LS2 9JT UK; 3 Department of Medical Psychology, Amsterdam UMC, VU University Medical Center, PO Box 7057, 1007 MB Amsterdam, The Netherlands; 4 Department of Neurology, Amsterdam UMC, VU University Medical Center, PO Box 7057, 1007 MB Amsterdam, The Netherlands; 5 Department of Otolaryngology – Head & Neck Surgery, Amsterdam UMC, VU University Medical Center, PO Box 7057, 1007 MB Amsterdam, The Netherlands; 6 Cancer Center Amsterdam, Amsterdam UMC, VU University Medical Center, PO Box 7057, 1007 MB Amsterdam, The Netherlands; 7 Brain Tumor Center Amsterdam, Amsterdam UMC, VU University Medical Center, PO Box 7057, 1007 MB Amsterdam, The Netherlands; 8 Amsterdam Public Health Research Institute, Amsterdam UMC, VU University Medical Center, PO Box 7057, 1007 MB Amsterdam, The Netherlands; 9 Clinical Psychology, VU University, Van der Boechorststraat 1, 1081 BT Amsterdam, The Netherlands

**Keywords:** Glioma, Family caregiver, Costs, Healthcare utilization, Work productivity, Depression

## Abstract

**Background:**

Gliomas are associated with significant healthcare burden, yet reports of costs are scarce. While many costs are unavoidable there may be treatable symptoms contributing to higher costs. We describe healthcare and societal costs in glioma patients at high risk for depression and their family caregivers, and explore relationships between costs and treatable symptoms.

**Methods:**

Data from a multicenter randomized trial on effects of internet-based therapy for depressive symptoms were used (NTR3223). Costs of self-reported healthcare utilization, medication use, and productivity loss were calculated for patients and caregivers separately. We used generalized linear regression models to predict costs with depressive symptoms, fatigue, cognitive complaints, tumor grade (low-/high-grade), disease status (stable or active/progression), and intervention (use/non-use) as predictors.

**Results:**

Multiple assessments from baseline through 12 months from 91 glioma patients and 46 caregivers were used. Mean overall costs per year were *M* = €20,587.53 (*sd* = €30,910.53) for patients and *M* = €5,581.49 (*sd* = €13,102.82) for caregivers. *In patients*, higher healthcare utilization costs were associated with more depressive symptoms; higher medication costs were associated with active/progressive disease. *In caregivers*, higher overall costs were linked with increased caregiver fatigue, cognitive complaints, and lower patient tumor grade. Higher healthcare utilization costs were related to more cognitive complaints and lower tumor grade. More productivity loss costs were associated with increased fatigue (all *P* < 0.05).

**Conclusions:**

There are substantial healthcare and societal costs for glioma patients and caregivers. Associations between costs and treatable psychological symptoms indicate that possibly, adequate support could decrease costs.

**Trial registration:**

Netherlands Trial Register NTR3223.

## Introduction

Gliomas cause not only a high disease burden that greatly affects patients’ and their family caregivers’ lives, but are also associated with a high healthcare burden [1]. Reports on direct (health and social care) and indirect (e.g., productivity loss due to work absence) costs are scarce and often not comprehensive, but hint towards costs being substantial [2, 3]. Individual indirect costs are higher compared to other brain disorders [3], and a small US study revealed substantial out-of-pocket costs among high-grade glioma patients (*N* = 43; median $1341 per month) [4]. These studies do not yet take into account social care and support costs often absorbed by family caregivers. Uncompensated caregiving hours do not only result in income loss (between €8124 and €20,196 annually) but also have tangible consequences for caregivers’ careers [5].

Depressive symptoms, fatigue, and cognitive deficits are common symptoms in glioma patients and may impact on patients’ and family caregivers’ quality of life [6–8]. But, these symptoms may also impact on direct and indirect costs. Depressive symptoms and fatigue have been linked with an increase in healthcare utilization and reduced work productivity [9–12]. Indeed, in working adults with malignant brain tumors (*N* = 131), depressive symptoms, fatigue, and cognitive issues accounted for 65% of the variance in work limitations [13]. Depressive symptoms, alongside cognitive issues, were predictive of difficulty performing at work and greater loss of productivity in patients with skull base tumors (*N* = 45) [14]. Despite the poor evidence-base for effectiveness of support in this population [15–17], depressive symptoms, cognitive deficits, and fatigue are potentially treatable symptoms.

Most brain tumor specific studies described above were carried out in the US, many did not link productivity loss to costs, none have presented patient and caregiver costs in one report, and none have investigated the relationship between costs and potentially reversible symptoms. We therefore aimed not only to describe costs in glioma patients and family caregivers, but also to explore associations between patients’ and caregivers’ depressive symptoms, fatigue, cognitive complaints and both patient and caregiver costs during a 12 month period. More knowledge on the costs associated with potentially treatable symptoms in a population with disproportionately high burden, will strengthen the case for further investigation of effectiveness of support programs to improve the lives of patients and family caregivers.

## Methods

### Participants

Patient-caregiver dyads were invited to participate in a Dutch nation-wide randomized controlled study to investigate the effectiveness of an internet-based guided self-help intervention in glioma patients with depressive symptoms [18, 19]. Recruitment procedures have been described in more detail elsewhere [18, 19]. Adult (> 18 years of age) glioma patients with WHO grade II, III or IV glioma, and at least mild depressive symptoms (CES-D score ≥ 12) were invited to participate. Exclusion criteria were (1) no access to the internet and/or no email address; (2) insufficient proficiency of the Dutch language; (3) suicidal intent. All patients were asked to invite a family caregiver (defined as the person providing the majority of mental and physical support) to participate. Participants were recruited between November 2011 and June 2015, with 95 of the 114 glioma patients expressing interest and meeting all inclusion criteria signing written informed consent (83.3%). Details on reasons for declining participation are not available.

### Procedures

Following consent procedures, assessments consisting of online questionnaires took place at baseline, 6 weeks, 12 weeks, 18 weeks, 24 weeks, 6 months, and 12 months [19]. Patients and caregivers reported on their own experiences. The study was approved by the institutional review board of the VU University Medical Center (Registration Number 2011/227), and was registered in the Netherlands Trial Registry (NTR3223).

### Outcome measures

The Trimbos/iMTA questionnaire for Costs associated with Psychiatric Illness (TIC-P) [20] was administered, which incorporates the Short-Form Health and Labor Questionnaire (SF-HLQ) [21]. It covers the direct costs of healthcare utilization from appointments and medication (including oral chemotherapy, but excluding procedures such as surgery and/or radiotherapy), and indirect costs due to productivity loss in three modules: absence from paid employment; production loss without absence from paid employment; and impediments to paid or unpaid employment. Further outcome measures included: the Epidemiological Studies-Depression Scale (CES-D) [22] for depressive symptoms; the Checklist Individual Strength (CIS) [23] for fatigue; the MOS cognitive functioning scale [24] for cognitive complaints; and the EORTC Brain Cancer Module [25] for disease-specific symptoms (patients only).

### Statistical analysis

All analyses were performed using STATA (v. 15) and SPSS software (v. 23). Medication costs per four weeks were calculated based on the Defined Daily Doses (DDDs) from the 2015 GIP databank, with €6 added per case to account for pharmacy prescription costs and a further 6% for taxes. Costs for healthcare utilization per four weeks were calculated based on the values published in the updated 2015 Cost Manual [26], plus travel and parking costs as appropriate. Costs for productivity loss (presenteeism and absenteeism per four weeks) were derived from the SF-HLQ, with €34.75 per working hour and €14 for each hour of unpaid work as recommended in the updated Cost Manual [26]. A total cost variable was calculated by adding costs for medication, healthcare utilization, and productivity loss. The inflator from 2015 to 2019 is 1.06 as per the Campbell and Cochrane Economics Methods Group/Evidence for Policy and Practice Information and Coordinating Centre Cost Converter [27], hence all costs were multiplied by 1.06.

Descriptive statistics were generated for sociodemographic characteristics, and baseline assessments of healthcare utilization, productivity loss, depressive symptoms (CES-D), fatigue (CIS), cognitive complaints (MOS), and costs in patient and caregiver groups. As cost data has a positively skewed distribution, we used generalized linear regression models (gamma family, log link) to identify statistically significant explanatory variables. We used multiple observations per participant (baseline, 6 weeks, 12 weeks, 18 weeks, 24 weeks, 6 months, 12 months) and robust standard errors based on participant to account for this. We included patients’ and caregivers’ self-reported depressive symptoms (CES-D), fatigue (CIS), and cognitive complaints (MOS) as potentially reversible symptoms. We included tumor grade (low-/high-grade) and disease status (stable/progression/active treatment) to cover important clinical differences within individuals. In patients only, intervention (use/non-use) was included to account for potential impact of the internet-based therapy—caregivers did not undergo any intervention. Variables associated at P < 0.10 and those of theoretical importance providing improvement in model fit were included in a multivariate model with backward selection to yield final prediction models, based on best fit according to the Akaike and Bayesian Information Criteria. We derived marginal effects for the included covariates and here, P < 0.05 was considered statistically significant. Missing data were not imputed, and no corrections for multiple testing were made due to the exploratory nature of the project.

## Results

### Participants

In total, 90 patients (291 observations) and 45 family caregivers (95 observations) completed at least one assessment and were included. Average age was 45 for patients and 48 for caregivers, see Table 1. More than half of patients were women (58.9%) and 53.5% of caregivers were men. 55.6% of patients were diagnosed with a WHO grade II glioma, and on average, patients were diagnosed 3.44 (*sd* = 4.84, range 0–26) years prior to enrolment. Nearly all caregivers were the patient’s spouse/partner (86.7%). Both patient and caregiver groups had relatively high baseline scores for depressive symptoms (patients *M* = 23.61, *sd* = 6.37; caregivers *M* = 17.16, *sd* = 6.34).

**Table 1:** Sociodemographic and clinical variables, depressive symptoms, fatigue, cognitive complaints, and mental wellbeing for both patients and caregivers

	Patients (*N* = 90)	Caregivers (*N* = 45)
Age^a^, M (sd), range	44.78 (11.81), 18–76	47.69 (11.40), 27–72
Sex^a^ N (%)		
Male	37 (41.1%)	24 (53.3%)
Female	53 (58.9%)	18 (40.0%)
Educational level^b^ N (%)		
Low	10 (11.1%)	6 (13.3%)
Middle	39 (43.3%)	15 (33.3%)
High	39 (43.3%)	23 (51.1%)
Other	2 (2.2%)	1 (2.2%)
Relationship status patient N (%)		
Not married	24 (26.7%)	–
Married or living together	59 (65.6%)	–
Divorced	7 (7.8%)	–
Relationship patient/caregiver^a^ N (%)		
Spouse or partner	–	39 (86.7%)
Family member	–	3 (675%)
Tumor type patient N (%)		
Pontine glioma	1 (1.1%)	0 (0%)
Ganglioglioma	1 (1.1%)	0 (0%)
Astrocytoma	39 (43.3%)	16 (35.6%)
Oligodendroglioma	22 (24.4%)	14 (31.1%)
Oligoastrocytoma	13 (14.4%)	7 (15.6%)
Glioblastoma	13 (14.4%)	8 (17.8%)
Unspecified glioma	1 (1.1%)	0 (0%)
WHO Grade N (%)		
II	50 (55.6%)	24 (53.3%)
III	26 (28.9%)	13 (28.9%)
IV	13 (14.4%)	8 (17.8%)
No grade	1 (1.1%)	0 (0%)
Time since patient’s diagnosis (years), M (sd), range	3.44 (4.84), 0–26	3.47 (4.65), 0–22
Disease-specific symptoms (BN20) M (sd)		
Future uncertainty	47.50 (20.99)	–
Visual disorder	20.49 (20.19)	–
Communication deficits	28.52 (24.02)	–
Headaches	30.37 (30.66)	–
Seizures	13.33 (22.79)	–
Drowsiness	31.11 (28.18)	–
Bothered by hair loss	14.07 (26.43)	–
Bothered by itching skin	19.62 (26.86)	–
Weakness of legs	10.00 (20.27)	–
Depressive symptoms (CES-D)	23.61 (6.37)	17.16 (6.34)
Fatigue (CIS)	93.73 (21.11)	59.09 (24.78)
Cognitive complaints (MOS) M (sd)	23.73 (6.37)	29.81 (4.14)
Comorbidities, N (%)		
Asthma, chronic bronchitis	6 (6.7%)	4 (8.9%)
Sinusitis	11 (12.2%)	6 (13.3%)
Heart disease	0 (0%)	3 (6.7%)
High blood pressure	10 (11.1%)	10 (22.2%)
Stroke	3 (3.3%)	0 (0%)
Serious bowel issues, longer than 3 months	3 (3.3%)	1 (2.2%)
Gallstones or gall bladder infection	0 (0%)	3 (6.7%)
Kidney stones	1 (1.1%)	0 (0%)
Prolaps	2 (2.2%)	0 (0%)
Diabetes	2 (2.2%)	0 (0%)
Thyroid issues	5 (5.5%)	1 (2.2%)
Chronic back issues	6 (6.7%)	3 (6.7%)
(Rheumatoid) arthritis	11 (12.2%)	7 (15.6%)
Migraine	12 (13.3%)	7 (15.6%)
Eczema	11 (12.2%)	4 (8.9%)

^a^Data from three caregivers missing

^b^Data from 1 caregiver missing

### Description of costs at baseline

#### Overall direct and indirect costs

At baseline, on average, the total direct and indirect costs per 4 weeks amounted to *M* = €1604.43 (*sd* = €2377.73) for patients and *M* = €429.34 (sd = €1007.91) for caregivers. On a yearly basis this translates to *M* = €20,857.53 (*sd* = €30,910.53) for patients and *M* = €5581.49 (*sd* = €13,102.82) for caregivers. This is broken down into separate costs for healthcare utilization, medication, and productivity loss as described below.

#### Healthcare utilization and medication

Healthcare service use per four weeks yielded a total per patient cost of *M* = €422.94 (*sd* = €912.84, see Table 2). Outpatient specialist care (33.3%) and GP services (31.1%) were accessed most regularly. Psychology or psychiatry services (other than the internet-based therapy tested), were used by 26.7% of patients. Home care services were used with highest frequency per service user (up to 56 contacts in four weeks, or twice a day). Medication was used by nearly all patients (90.0%) and was associated with an average cost of €107.95 (*sd* = €203.43, range €0–€1148.14) per four weeks. The majority of patients (77.8%) used antiepileptic drugs (AEDs), for which the DDD ranged between €0.11 (phenytoin) to €5.38 (lacosamide), with many taking multiple drugs concurrently. Some patients were taking chemotherapeutic drugs, often in combination (12.2%) for which the DDD ranged between €3.88 (lomustine) and €17.63 (vincristine).

**Table 2:** Healthcare utilization, medication costs and productivity loss per four weeks

Healthcare utilization	Patients *N* = 90			Caregivers *N* = 45		
	N (%) using service	# Contacts M (sd), range	M (sd) costs	N (%) using service	# Contacts M (sd), range	M (sd) costs
General practitioner	28 (31.1%)	1.76 (1.12), 1–6	€61.60 (40.22)	10 (21.2%)	1.4 (0.52), 1–2	€49.28 (18.18)
Company doctor	13 (14.4%)	1.31 (0.63), 1–3	€75.25 (36.28)	2 (4.4%)	1.5 (0.71), 1–2	€86.32 (40.69)
Outpatient specialist care	30 (33.3%)	2.67 (2.12), 1–10	€269.47 (214.50)	6 (13.3%)	1.5 (0.84), 1–3	€151.57 (84.54)
Inpatient specialist care	1 (1.1%)	4 (–), N/a	€2036.60 (N/a)	1 (2.2%)	1 (-), N/a	€509.15 (N/a)
Psychology/psychiatry services (private practice)	7 (7.7%)	2.25 (1.28), 1–4	€237.32 (143.29)	3 (6.7%)	2.33 (2.31), 1–5	€242.26 (239.78)
Psychology/psychiatry services (mental healthcare center)	1 (1.1%)	4 (–), N/a	€440.32 (N/a)	2 (4.5%)	3.5 (2.12), 2–5	€385.28 (233.52)
Psychology/psychiatry services (outpatient hospital)	14 (15.6%)	2.00 (1.92), 1–8	€216.94 (208.43)	4 (8.9%)	1 (0), N/a	€108.47 (0)
Psychology/psychiatry services (day case hospital)	2 (2.2%)	8.5 (10.61), 1–16	€2773.73 (3461.16)	0 (0%)	N/a	N/a
Physiotherapist	14 (15.6%)	5.21 (3.53), 1–12	€184.71 (125.18)	3 (6.7%)	3.67 (3.79), 1–8	€129.88 (134.11)
Social worker	6 (6.7%)	2.17 (1.17), 1–4	€151.47 (81.72)	3 (6.7%)	2.00 (1.73), 1–4	€139.81 (121.08)
Alternative healer	5 (5.6%)	1.60 (0.89), 1–3	€130.42 (72.91)	0 (0%)	N/a	N/a
Self-help	3 (3.3%)	3.33 (4.04), 1–8	€207.58 (251.68)	1 (2.2%)	2 (-), N/a	€124.55 (N/a)
Home care	7 (7.7%)	18.79 (17.19), 3.5–56	€995.64 (911.31)	1 (2.2%)	22.00 (-), N/a	€1166.00 (N/a)
Total healthcare utilization costs	*N* = 84		€422.94 (912.84)	*N* = 43		€142.21 (397.01)
Medication costs	*N* = 89		€107.95 (203.43)	*N* = 42		€14.13 (22.54)

Productivity	N (%)		N (%)	
In paid employment	34 (37.4%)		30 (66.7%)	
Not in paid employment	51 (56.0%)		12 (26.6%)	
Absenteeism	44 (48.9%)	€2363.29 (2760.83)	11 (24.4%)	€1174.48 (1355.82)
Presenteeism	7 (7.7%)	€505.17 (424.12)	3 (6.7%)	€417.46 (297.73)
Total productivity loss	*N* = 85	€1264.95 (2299.97)	*N* = 42	€337.42 (901.15)
Overall total costs	*N* = 78	€1604.43 (2377.73)	*N* = 39	€429.34 (1007.91)

For caregivers, average costs associated with healthcare service use per four weeks were *M* = €142.21 (*sd* = €397.01). About a fifth of caregivers (21.2%) had visited their GP, and 13.3% had attended an outpatient hospital appointment. Psychology or psychiatry services were accessed by 20.0% of caregivers. Home care services were used with highest frequency per service user (up to 22 visits in four weeks). Medication was used by 48.9% of caregivers with an average cost of €14.13 (*sd* = €22.54) per four weeks.

#### Productivity loss

At baseline, over a third of patients reported to be in paid employment (37.4%). Others described their productivity status as being declared unfit for work (in whole or in part, 41.1%), taking primary care of the household (13.3%), retired (5.6%), student (3.3%), or ‘other’ (11.1%). Many reported to be hindered by health problems in everyday tasks: domestic work (54.4%), grocery shopping (45.6%), chores around the house (44.4%), and taking care of children (24.4%). Absenteeism was reported by 48.9% of patients, and presenteeism (productive hours lost while working) by another 7.8%. Together, the costs for productivity loss were *M* = €1264.95 (*sd* = €2299.97) per patient (employed and unemployed) per four weeks.

Most caregivers reported they were in paid employment at baseline (66.7%). Others were retired (13.3%), declared unfit for work (in whole or in part, 6.7%), taking primary care of the household (2.2%), or reported ‘other reasons’ (4.4%). A minority of caregivers reported to have been hindered by health issues in domestic work (6.7%), grocery shopping (8.8%), chores (6.7%), or child care (4.4%). Absenteeism was reported by 24.4% of caregivers, and presenteeism by another 6.7%. In total, productivity loss in caregivers was on average €337.42 (*sd* = €901.15) per four weeks.

### Associations between costs and treatable psychological symptoms

#### Glioma patients

All significant associations based on univariate and multivariate analysis in patients are shown in Table 3. In univariate analysis of healthcare costs in patients, there were significant associations between increased healthcare utilization costs and increased symptoms of depression (+€18.34 per four weeks with each unit increase in scores); fatigue (+€5.49); and cognitive complaints (+€6.17; all *P* < 0.05). In the final multivariate model which included depressive symptoms and tumor grade, depressive symptoms remained significantly associated with increased healthcare costs (+€24.46, *P* = 0.001).

**Table 3:** Associations between patient costs and patients’ depressive symptoms, cognitive complaints, fatigue, and clinical characteristics

Model		Margins	95% CI	P-value	No Obs
Costs	Predictors				
Univariate					
Healthcare utilization costs	Depressive symptoms	18.343	1.026 to 35.659	0.009**	270
	Fatigue	5.491	− 0.774 to 11.756	0.050**	268
	Cognitive complaints	6.174	0.297 to 12.051	0.019**	266
Medication costs	Depressive symptoms	− 2.481	− 5.411 to 0.450	0.053**	278
	Cognitive complaints	− 1.889	− 3.165 to − 0.614	< 0.001**	276
	Tumor grade:			0.015**	279
	Low-grade	53.299	31.152 to 75.447		
	High-grade	109.826	65.023 to 154.628		
	Disease status:			< 0.001**	262
	Stable	46.422	32.404 to 60.440		
	Active/progression	164.319	92.078 to 236.500		
Multivariate					
Healthcare utilization costs	Depressive symptoms	24.459	3.662 to 45.250	0.001**	270
	Tumor grade	182.878	− 96.438 to 462.190	0.170	
Medication costs	Cognitive complaints	− 0.694	− 1.505 to 0.117	0.078*	259
	Tumor grade	31.619	− 5.489 to 68.727	0.078*	
	Disease status	76.171	29.580 to 122.0761	< 0.001**	

^*^P < 0.10; **P < 0.05

In univariate analysis of medication costs, there were significant associations between higher medication costs and decreased depressive symptoms (−€2.48 per 4 weeks with each unit increase in scores), fewer cognitive complaints (−€1.89), higher tumor grade (+€56.53 for high-grade glioma vs. low-grade glioma), and active disease status (+€117.90 for active disease or progression vs. stable disease; all *P* < 0.05). The final multivariable model included cognitive complaints, tumor grade and disease stage. Active/progressive disease remained a statistically significant predictor of medication costs (+€76.17 for active/progressive disease vs. stable disease, *P* < 0.001).

#### Family caregivers

All significant associations based on univariate and multivariate analysis in caregivers are shown in Table 4. In univariate analysis of overall costs, there were significant associations between higher costs and increased caregiver-reported depressive symptoms (+€14.57), fatigue (+€12.25), cognitive complaints (+€18.29), and patient tumor grade (+€253.72 for low-grade glioma vs. high-grade glioma; all *P* < 0.10). In the final multivariate model, higher overall costs were associated with increased fatigue (+€5.60) and lower patient tumor grade (−€384.95; all *P* < 0.05).

**Table 4:** Associations between caregiver costs and caregivers’ depressive symptoms, cognitive complaints, fatigue, and patient clinical characteristics

Model		Margins	95% CI	P value	No obs
Costs	Predictors				
Univariate					
Healthcare utilization costs	Depressive symptoms	8.029	− 0.014 to 16.071	< 0.001**	93
	Fatigue	3.987	− 2.706 to 10.680	< 0.003**	94
	Cognitive complaints	5.565	− 1.198 to 12.329	0.003**	94
	Tumor grade:			0.009**	94
	Low-grade	147.822	12.170 to 283.473		
	High-grade	34.389	12.584 to 56.193		
Medication costs	Depressive symptoms	− 1.152	− 2.347 to 0.043	0.019**	91
Productivity loss costs	Depressive symptoms	12.798	2.338 to 23.258	0.031**	89
	Fatigue	21.658	− 17.113 to 60.429	0.002**	89
	Cognitive complaints	16.008	− 9.646 to 41.661	0.043**	89
	Tumor grade:			0.061*	89
	Low-grade	341.892	28.323 to 655.461		
	High-grade	114.327	34.034 to 194.620		
Overall total costs	Depressive symptoms	14.567	2.612 to 26.520	0.030**	83
	Fatigue	12.252	0.085 to 24.419	< 0.001**	83
	Cognitive complaints	18.288	− 6.768 to 43.344	0.027**	83
	Tumor grade:			0.088*	83
	Low-grade	430.613	67.937 to 793.290		
	High-grade	176.897	69.767 to 284.026		
Multivariate					
Healthcare utilization	Fatigue	0.965	− 0.518 to 10.223	0.176	94
	Cognitive complaints	4.803	− 0.617 to 10.224	0.006**	
	Tumor grade	− 125.810	− 244.069 to − 7.552	0.001**	
Productivity loss costs	Fatigue	14.021	− 10.945 to 38.988	0.020**	89
	Cognitive complaints	20.426	− 23.570 to 64.422	0.105	
	Tumor grade	− 679.380	− 2132.796 to 774.037	0.051*	
Overall total costs	Fatigue	5.596	0.024 to 11.980	0.034**	83
	Cognitive complaints	14.334	− 3.765 to 32.433	0.052*	
	Tumor grade	− 384.950	− 733.918 to -35.980	0.003**	

^*^P < 0.10; **P < 0.05

In univariate analysis of healthcare utilization costs, there were significant associations with increased caregiver-reported depressive symptoms (+€8.03 per 4 weeks with each unit increase in scores), fatigue (+€3.99), cognitive complaints (+€5.57), and lower patient tumor grade (−€113.43 for high-grade glioma vs. low-grade glioma; all *P* < 0.05). In the final multivariate model which included cognitive complaints, fatigue, and tumor grade, only increased caregiver-reported cognitive complaints (+€4.80) and lower tumor grade (−€125.81) remained statistically significantly associated with higher healthcare costs (*P* < 0.05).

Medication costs were significantly associated with lower caregiver depression scores (−€1.15, *P* = 0.019) only.

In univariate analysis, higher productivity loss costs were significantly associated with increased caregiver-reported depressive symptoms (+€12.80), fatigue (+€21.66), cognitive complaints (+€16.01), and patient tumor grade (−€227.57 for high-grade glioma vs. low-grade glioma; all *P* < 0.10). The final multivariate model included fatigue, cognitive complaints, and tumor grade: only fatigue (+€5.60) and lower tumor grade (−€384.95) remained significantly associated with increased productivity loss costs (P < 0.05).

## Discussion

Very little is known about the true costs of primary brain tumors for patients, caregivers, the healthcare system and society. Our study is one of the first to provide meaningful quantitative data by focusing on the costs associated with self-reported healthcare utilization, medication, and productivity loss of glioma patients and caregivers. The healthcare costs measured excluded most anti-tumor procedures, but included adjuvant chemotherapy—thus essentially reflect maintenance costs only. We found that on average 3 years after diagnosis, healthcare utilization, medication use, and productivity loss resulted in yearly costs of *M* = €20,857.53 in glioma patients at high risk for depression; with an additional yearly cost of *M* = €5581.49 in family caregivers. Overall yearly costs per dyad (€26,439.02) approaches the yearly Dutch median income per household [28].

Costs varied greatly between participants as evidenced by the large standard deviations. Interestingly, while only 1/3rd of patients and 2/3rds of caregivers were in paid employment, productivity loss costs accounted for > 78% of costs. Of note, most costs are not directly absorbed by patient-caregiver dyads. Rather, costs are divided evenly across the Dutch population through the social security system, and thus reflect a societal cost rather than a personal cost.

Many consequences of brain tumors are unavoidable or difficult to manage. We therefore investigated associations between those symptoms that are potentially reversible (depressive symptoms, fatigue, cognitive complaints) and costs. Despite being a patient-caregiver group at high risk for depression, less than a quarter of participants had accessed psychology/psychiatry services. In patients, reversible symptoms were not very strongly related to costs: in multivariate analyses, increased healthcare costs were associated with increased depressive symptoms. The association between medication costs and cognitive complaints approached statistical significance. In part, this could be explained by the relative homogeneity of our patient sample, as all were at increased risk of depression. Previous studies found associations between depressive symptoms, fatigue, cognitive complaints, and work limitations [13, 14]. Work limitations reflect a person’s experienced difficulty in managing time and demands rather than costs from presenteeism/absenteeism—which does not necessarily overlap.

In caregivers, higher overall costs were linked with increased fatigue and cognitive complaints; higher healthcare utilization costs were related to more cognitive complaints; and higher productivity loss costs were associated with increased fatigue. As caregivers did not suffer from a direct physical cause of cognitive complaints, these may have been an indirect consequence of increased burden. Our results seem to confirm the large role of fatigue in work productivity found in the general working age population [12]. Interestingly, depressive symptoms seemed to impact less than expected [9–11]. Some caution is warranted when interpreting these findings, as these are based on a small sample size, and in some cases show statistical significance despite having 0 in the 95% confidence interval. Lower tumor grade was predictive of higher costs in our caregiver sample. Compared to patients with more aggressive brain tumors, low-grade glioma patients survive for longer, more frequently suffer from epilepsy, and are relatively young with often young families and a busy working life. Our findings confirm the high social impact and financial toxicity of low-grade glioma from the caregiver perspective. Helping caregivers cope better can potentially reduce overall costs associated with brain tumors by over 20%. If not properly supported, costs could increase over time as prolonged periods of stress and poorer self-care in caregivers can lead to poor physical health effects [29, 30].

## Strengths and limitations

Strengths of the current study are the detailed self-reported use of healthcare services, medications, and productivity loss parameters; the longitudinal nature of the data; and our robust modelling techniques. Our analyses are exploratory and not corrected for multiple testing. The self-report nature of the data may have introduced some bias, leading to over- or underestimation of costs. For example, productivity loss costs are based on self-reported absence or reduced productivity—which may not completely overlap with an employer’s records. Finally, our patients were at high risk for depression, thus findings may not be generalizable to all glioma patient-caregiver populations. However, the RCT was intended to have high external validity and thus few exclusion criteria were employed. This is reflected in a relatively heterogeneous sample of glioma patients in terms of grade, localization, and treatment, as well as the reasons for attrition (which include e.g., cognitive issues, relief of symptoms, and disease progression [18]).

## Conclusions

Nevertheless, we were able to show that potentially treatable symptoms are linked with higher healthcare utilization costs in patients, and higher overall costs, healthcare utilization costs, and productivity loss in caregivers. More generally, we demonstrated that healthcare service and medication use, and productivity loss costs for patient-caregiver dyads are substantial and vary greatly between individuals. This study provides evidence that the true cost of brain tumors is a burden shared between patients, caregivers, the healthcare system, and society as a whole.
